# Effective treatment of malignant atrophic papulosis (Köhlmeier-Degos disease) with treprostinil – early experience

**DOI:** 10.1186/1750-1172-8-52

**Published:** 2013-04-04

**Authors:** Lee S Shapiro, Aixa E Toledo-Garcia, Jessica F Farrell

**Affiliations:** 1 Steffens Scleroderma Center, Saratoga Springs, NY, USA; 2 The Center for Rheumatology, NY, USA; 3 Albany College of Pharmacy and Health Sciences, Albany, NY, USA

**Keywords:** MAP, Treprostinil, Malignant atrophic papulosis

## Abstract

**Background:**

Malignant atrophic papulosis (Köhlmeier-Degos disease; MAP) is an uncommon endotheliopathy with pathological findings similar to the vascular lesions of systemic sclerosis. These two disorders can overlap. When associated with visceral lesions, MAP has been considered almost universally and rapidly fatal. A recent report described dramatic response to treatment with eculizumab, but disease progression after initial response to therapy has occurred.

**Methods:**

We describe the clinical and pathologic findings in two patients, one with MAP and the other with MAP like lesions, who received treatment with subcutaneous treprostinil. One patient had an overlap syndrome with features of systemic lupus erythematosus (SLE) and scleroderma and severe pulmonary hypertension. She also had very extensive MAP like cutaneous lesions. There was no evidence of central nervous system (CNS) disease and laparoscopy revealed no visible MAP lesions on the serosa of the small bowel. The second patient had experienced life-threatening disease progression despite ongoing eculizumab therapy. During this treatment, he had developed CNS and bladder involvement with neurologic symptoms and gross hematuria.

**Results:**

Patient one was placed on therapy with treprostinil for her pulmonary hypertension, but in the months subsequent to initiation of treatment, dramatic and complete resolution of cutaneous MAP like lesions and disabling digital pain occurred. In patient two, therapy with treprostinil was temporally associated with clearing of hematuria, resolution of CNS symptoms and improvement in MRI findings.

**Conclusions:**

Treprostinil may offer a second effective treatment approach to individuals with MAP or “rescue therapy” to those in whom eculizumab treatment has failed to maintain suppression of disease activity.

## Background

Malignant atrophic papulosis (Köhlmeier-Degos disease; heretofore referred to as MAP) is a rare vasculopathy presenting with avascular skin lesions, often with a telangiectatic rim. Although cases of skin-only involvement in MAP are probably under-reported in the literature, as many as 85% of individuals described in case reports progressed within a few years to systemic disease. MAP is a non-inflammatory vasculopathy affecting most commonly the gastrointestinal tract (especially the jejunum), also often the central nervous system, pericardium, and bladder [1,2]. The etiopathogenesis of MAP is unknown [3]. Once systemic, the disease is almost universally fatal within two to three years [2,4]. Death most commonly results from repeated gastrointestinal perforations and sepsis, but can also be the consequence of neurologic events and cardiac disease. The vascular pathology is strikingly similar to changes of scleroderma with marked intimal fibrosis progressing to obliteration or near obliteration of vessel lumen [4]. Cutaneous lesions consistent with MAP can occur in individuals with scleroderma, dermatomyositis, and lupus [5-7].

Treprostinil, a prostacyclin analog, causes direct vasodilation of pulmonary and systemic arterial beds as well as inhibition of platelet aggregation. Recent data suggests that treprostinil may enhance the number and function of endothelial progenitor cells (EPC) in pediatric pulmonary hypertension patients [8].

## Results

### Patient one

Patient one is a now 42-year-old woman initially seen in 2008. She presented then with a three year history of cold-induced blanching of her hands and feet, retrosternal heartburn of similar duration, with esophageal dysmotility on barium swallow. Anti-nuclear antibody (ANA) was present at a titer of 1:640 in anti-nucleolar pattern. Anti-topoisomerase antibody, anti-U1 RNP antibody, SS-A, and SS-B antibodies were negative.

In the year prior to her presentation, she developed alopecia, lymphadenopathy, and autoimmune hemolytic anemia. Bone marrow demonstrated hypercellular marrow with polyclonal T-cell lymphocytes, mild dyserythropoiesis, and dysgranulopoiesis. She was placed on prednisone and mycophenolate. When seen, she had 1+ digital and facial skin thickening on a Rodnan skin score of 0 to 3+. She also presented with extensive avascular lesions with telangiectatic borders at her digits and hands, upper arms, chest, and toes (Additional files 1, 2, 3). Skin biopsy of upper arm lesion was initially read as “consistent with lupus”. Proton pump inhibitor and hydroxychloroquine were added to her drug regimen. The skin lesions increased in extent and chloroquine was substituted for hydroxychloroquine without benefit. In early 2009, she developed pericarditis and pericardial tamponade and had a pericardial window placed. She also required cholecystectomy because of gangrenous cholecystitis. Cardiac catheterization post surgery demonstrated mean pulmonary artery (PA) pressure of 32/10. She experienced a grand mal seizure and was placed on Keppra. Mycophenolate was withdrawn and she received monthly pulse cyclophosphamide for six months and then was placed on azathioprine. Profound hypocomplementemia resolved, anti-DNA antibody, previously modestly elevated, disappeared.

She developed progressive dyspnea and in early 2010 on echocardiogram revealed estimated PA systolic pressure of 58. Right heart catheterization demonstrated mean PA pressure of 36.

Skin lesions, resembling clinically and histologically those of MAP, continued to increase in number. She also was experiencing frequent abdominal pain. Laparoscopy was performed but no serosal lesion suggestive of MAP was seen and her abdominal pain spontaneously abated. Therapy with subcutaneous treprostinil was initiated in August 2010 and titrated over a few months to 38.75 ng/kg/min. Within a month, she noted improved functional status with less dyspnea. She reported less discomfort at her hands and toes (she had been requiring fentanyl 75 mcg patches and hydromorphone 2 mg 2–3 times per day). Within three months of initiating therapy, she noted that the digital lesions were diminishing in size and the lesions on her upper arms and inframammary areas showed marked involution. No new lesion developed. Her World Health Organization (WHO) functional status improved to class II. She had sustained improvement so that by six months of therapy, the digital lesions more or less completely resolved and the toe lesions resolved soon thereafter. Residual scarring has persisted at the upper arms and inframammary area (Additional files 1, 2, 3). She has had no new neurologic event or any recurrence of abdominal pain.

### Patient two

Patient two, a 17-year-old male, was first seen in our clinic in August 2009. He had a two year history of an increasing number of avascular slightly depressed porcelain like lesions with an erythematous rim, primarily on his chest and abdomen, with more scattered lesions on his limbs, but none on his head, back or genitalia. Biopsy of a truncal lesion was consistent with a diagnosis of MAP (Additional file 4). He had no clinical or serologic findings to suggest overlap with any connective tissue disorder. Colonoscopy revealed a limited number of similar appearing lesions on the bowel wall. Within a few months, he started to experience episodes of crampy abdominal pain. These increased in frequency and intensity and he developed an acute abdomen in December 2009. At laparotomy, the serosa of the small bowel revealed hundreds of lesions consistent with MAP (Additional file 5). He had peritoneal fluid with polymicrobial growth but no area of bowel perforation was identified. He was treated with broad spectrum antibiotic therapy but redeveloped evidence of acute abdomen and required re-exploration 10 days later. He was found to have edematous and ischemic appearing loops of bowl with “abdominal compartment syndrome;” but again, no frank perforation. The abdominal wound was left open. He remained febrile, hypertensive, and tachycardic. He was treated with IV eculizumab based on successful use of this agent in a patient similarly afflicted [9-11]. Almost immediate improvement was noted, with resolution of fever and tachycardia and a decrease in swelling of the bowel loops. On continued eculizumab, he had sustained improvement and the abdominal wound was successfully closed after another 10 days.

He was able to return to school within two months without abdominal pain.

Despite continued eculizumab therapy, by late 2010 he had redeveloped bouts of abdominal pain and he had developed gross hematuria with cystoscopy showing lesions suspicious for MAP. The abdominal pain was of such severity as to require bowel rest and parenteral nutrition. Eculizumab dose was increased at this time hoping for better tissue penetration. Peak and trough levels of eculizumab were adequate to assure complete suppression of C5 activation. He also developed evidence of CNS disease with intermittent episodes of aphasia and arm numbness with new abnormalities on MRI consistent with findings described in other individuals with MAP (Additional file 6). At this point, the frequency of eculizumab was increased.

Based on our experience with patient one, who by this time had shown clearing of most of her skin lesions, we were able to obtain approval for subcutaneous treprostinil therapy on a compassionate use basis and it was initiated on December 28, 2010 and titrated upwards.

Due to continued abdominal pain, on January 24, 2011, he underwent laparoscopic surgery. Multiple adhesions were identified and lysed, but all previously noted serosal lesions of MAP had resolved (the surgeon was the same surgeon who had operated upon him in 2009.) Since that time, he has had no further abdominal pain and has gained weight progressively. He noted gradual involution of skin lesions, although these responded less rapidly and dramatically than those in patient one. He has had no further CNS event and repeated MRI showed improvement (Additional file 6). At last examination he had no neurologic deficit. He had no further gross or microscopic hematuria. Repeat cystoscopy was not performed. His current treprostinil dose is 32 ng/kg/min. He is attending college full-time and working in a supermarket part-time.

## Discussion

MAP has vascular pathology similar to that of scleroderma but the disease phenotype and pattern of organ involvement differ markedly from that of systemic sclerosis. Nevertheless, overlap of MAP and systemic sclerosis in addition to overlap of MAP with dermatomyositis and lupus have been described [4-6]. Our first patient had clinical and laboratory features of both systemic sclerosis and lupus. Our second patient had “primary MAP”.

In both patients, dramatic and sustained improvement in clinical status occurred following initiation of treprostinil, though the response was not immediate. In patient one, a decrease in digital pain occurred before involution and then resolution of the skin lesions occurred. The process of clearing the skin lesions took some months. In patient two, cutaneous response was less dramatic and slower, but treprostinil therapy coincided with resolution of neurologic symptoms and improvement in MRI. Laparotomy revealed complete clearing of the bowel lesions. Laparoscopic images were not obtained just prior to initiation of the treprostinil, but it is unlikely the eculizumab resulted in clearing of lesions as during the course of eculizumab, bladder lesions and CNS lesions developed.

Trepostinil therapy resulted in dramatic and sustained improvement in a patient with MAP and another with scleroderma/lupus overlap and MAP like lesions. Further clinical trials are warranted to confirm that treprostinil is an effective therapy for what has been a progressive, untreatable, and generally rapidly fatal disorder. The mechanism of action of treprostinil in this setting is unclear. MAP, like scleroderma, may be associated with a failure of angiogenesis. There are no studies on EPC populations in MAP. However, if they are suppressed, it may be that treprostinil can increase these cell populations, promoting angiogenesis. Treprostinil was recently reported to have this effect on EPC populations in pediatric patients with pulmonary arterial hypertension [8].

## Competing interests

The authors declare that they have no competing interests.

## Authors’ contributions

LS, AT and JF wrote the manuscript. LS, AT and JF read, revised and approved the final manuscript.

## Supplementary Material

Additional file 1 Left: Patient 1- extensive avascular lesions with telangectatic borders at her digits and hands before treatment with treprostinil Right: Patient one - after treatment with treprostinil. Click here for file

Additional file 2 **Left-Patient one lower extremity before treatment with treprostinil.** Right-patient one lower extremity after treatment with treprostinil. Click here for file

Additional file 3 **Left: Patient one-upper extremity with typical Degos like lesions before treatment with treprostinil.** Right: Patient one-upper extremity after treatment with treprostinil. Click here for file

Additional file 4 **Patient Two skin biopsy with typical histology: wedge-shaped area of infarction, epidermal atrophy with collagen degradation.** Vascular endothelial proliferation. In this case contain fibrin deposition. (Commonly thrombus is also present) Click here for file

Additional file 5 **Patient Two with multiple typical Degos lesions on small bowel and skin in December 2009.** On repeat laparotomy one month after initiation of treprostinil, bowel lesions appeared to have completely resolved. Click here for file

Additional file 6 **Patient Two-A: MRI imaging shows the T1 post-contrast image demonstrating the cord lesion in the left frontal lobe in 12/2010.** B: On repeat imaging in 07/2011, the enhancemmostly resolved. Click here for file
