# Supplementary material for: Effective treatment of malignant atrophic papulosis (Köhlmeier-Degos disease) with treprostinil – early experience
Source: Orphanet J Rare Dis. 2013 Apr 4;8:52. doi: 10.1186/1750-1172-8-52 (PMC3636001; doi:10.1186/1750-1172-8-52)
Supplement: Additional file 1 — Left: Patient 1- extensive avascular lesions with telangectatic borders at her digits and hands before treatment with treprostinil Right: Patient one - after treatment with treprostinil. [file 1750-1172-8-52-S1.pdf]

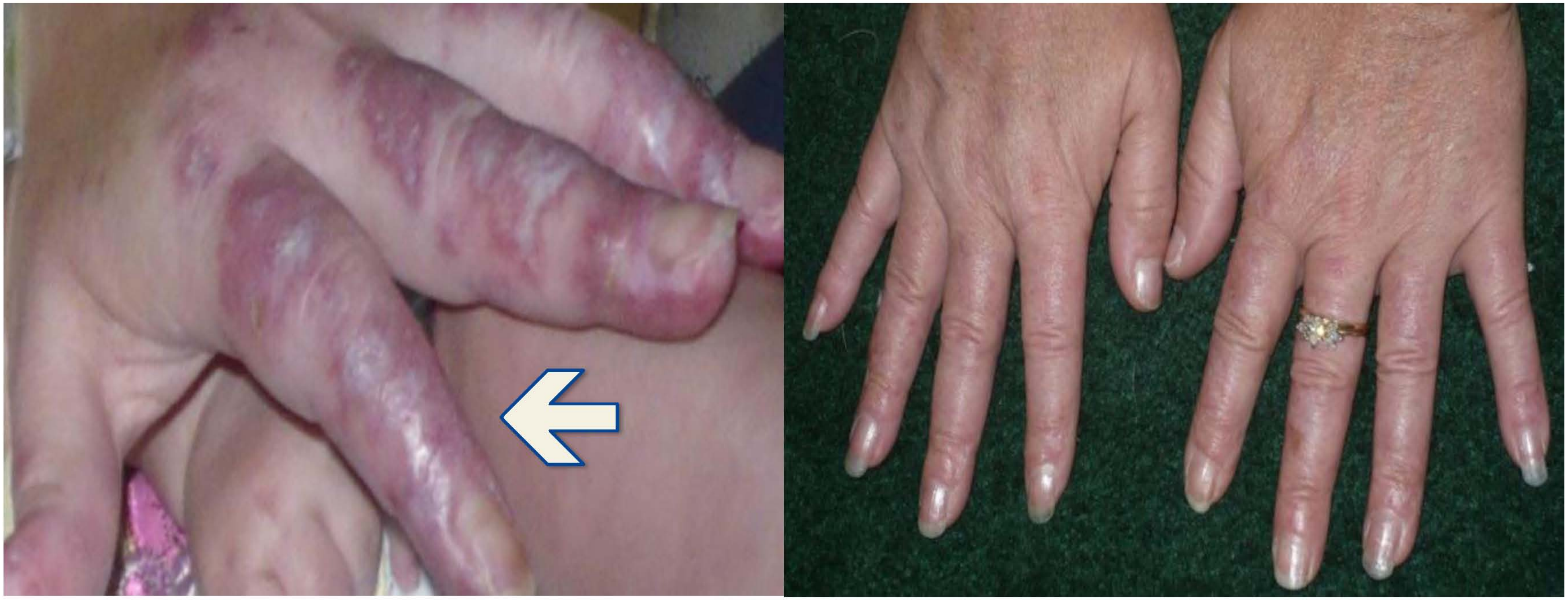

(Image One)

Left: Patient 1 – extensive avascular lesions with telangectatic borders at her digits and hands before treatment with treprostinil Right: Patient one – after treatment with treprostinil.
