# Supplementary material for: Effective treatment of malignant atrophic papulosis (Köhlmeier-Degos disease) with treprostinil – early experience
Source: Orphanet J Rare Dis. 2013 Apr 4;8:52. doi: 10.1186/1750-1172-8-52 (PMC3636001; doi:10.1186/1750-1172-8-52)
Supplement: Additional file 2 — Left-Patient one lower extremity before treatment with treprostinil. Right-patient one lower extremity after treatment with treprostinil. [file 1750-1172-8-52-S2.pdf]

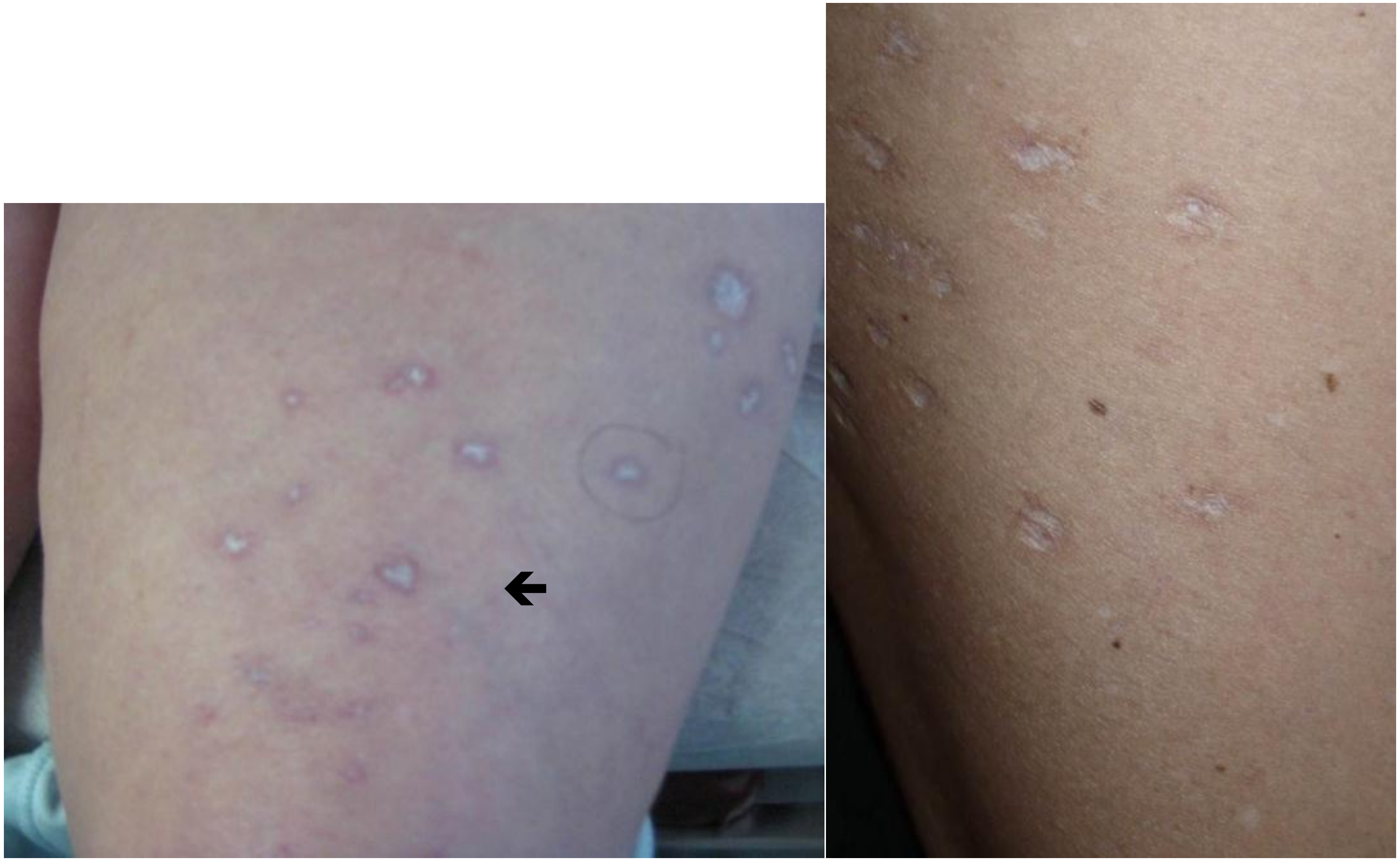

(Image Two)

Left – Patient one lower extremity before treatment with treprostinil. Right – patient one lower extremity after treatment with treprostinil.
