# Supplementary material for: Effective treatment of malignant atrophic papulosis (Köhlmeier-Degos disease) with treprostinil – early experience
Source: Orphanet J Rare Dis. 2013 Apr 4;8:52. doi: 10.1186/1750-1172-8-52 (PMC3636001; doi:10.1186/1750-1172-8-52)
Supplement: Additional file 4 — Patient Two skin biopsy with typical histology: wedge-shaped area of infarction, epidermal atrophy with collagen degradation. Vascular endothelial proliferation. In this case contain fibrin deposition. (Commonly thrombus is also present) [file 1750-1172-8-52-S4.pdf]

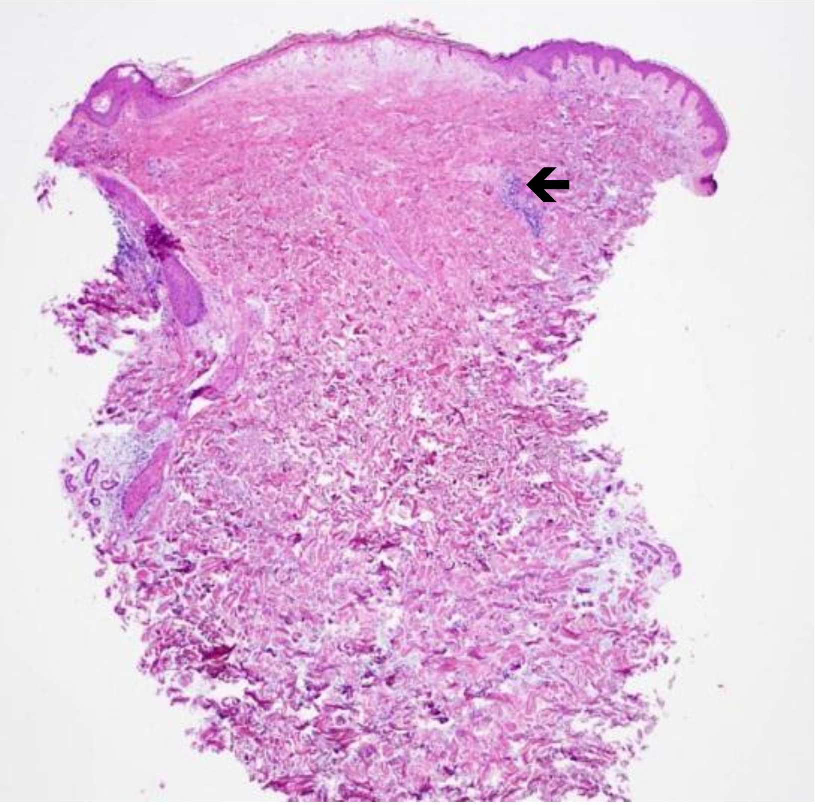

(Image Four)

Patient Two skin biopsy with typical histology: wedge-shaped area of infarction, epidermal atrophy with collagen degradation. Vascular endothelial proliferation. In this case contain fibrin deposition. (Commonly thrombus is also present)
