# Supplementary material for: Effective treatment of malignant atrophic papulosis (Köhlmeier-Degos disease) with treprostinil – early experience
Source: Orphanet J Rare Dis. 2013 Apr 4;8:52. doi: 10.1186/1750-1172-8-52 (PMC3636001; doi:10.1186/1750-1172-8-52)
Supplement: Additional file 5 — Patient Two with multiple typical Degos lesions on small bowel and skin in December 2009. On repeat laparotomy one month after initiation of treprostinil, bowel lesions appeared to have completely resolved. [file 1750-1172-8-52-S5.pdf]

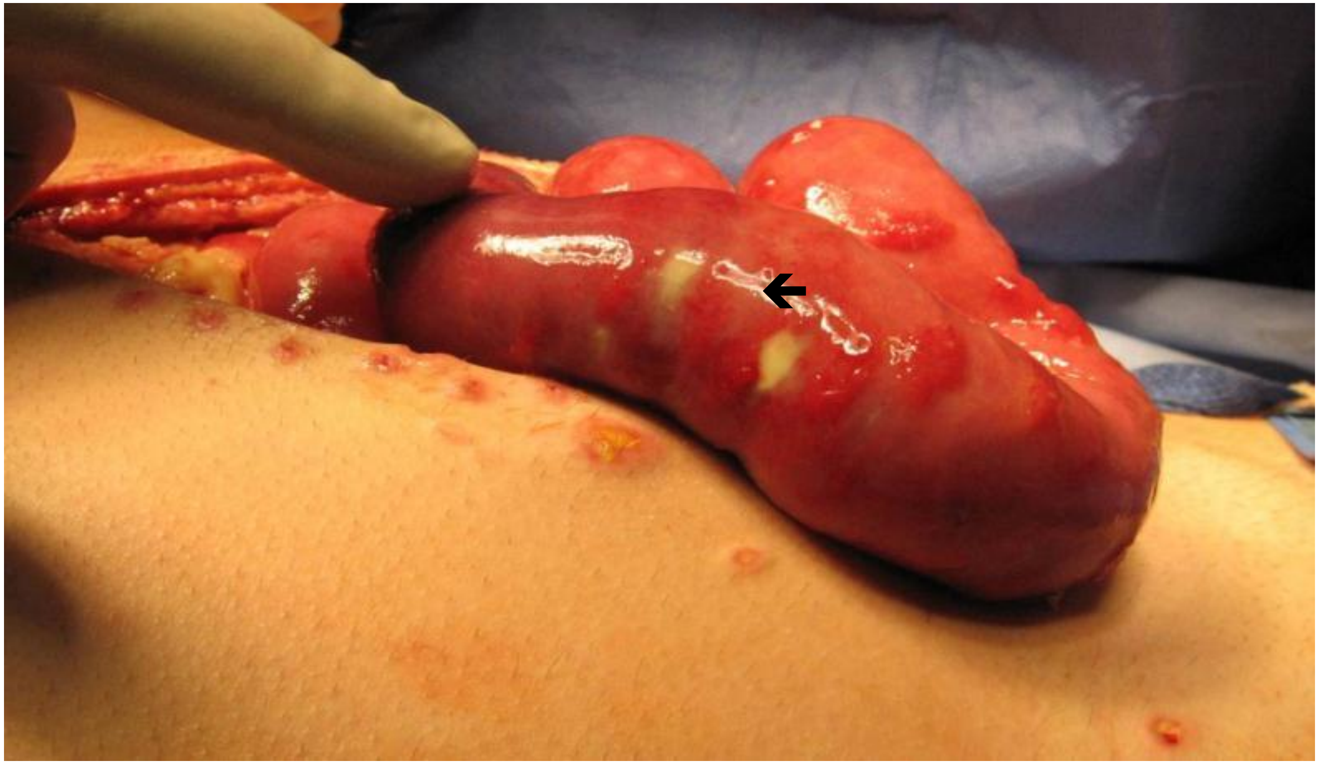

(Image Five)

Patient Two with multiple typical Degos lesions on small bowel and skin in December 2009. On repeat laparotomy one month after initiation of treprostinil, bowel lesions appeared to have completely resolved.
