# Supplementary material for: Effective treatment of malignant atrophic papulosis (Köhlmeier-Degos disease) with treprostinil – early experience
Source: Orphanet J Rare Dis. 2013 Apr 4;8:52. doi: 10.1186/1750-1172-8-52 (PMC3636001; doi:10.1186/1750-1172-8-52)
Supplement: Additional file 6 — Patient Two-A: MRI imaging shows the T1 post-contrast image demonstrating the cord lesion in the left frontal lobe in 12/2010. B: On repeat imaging in 07/2011, the enhancemmostly resolved. [file 1750-1172-8-52-S6.pdf]

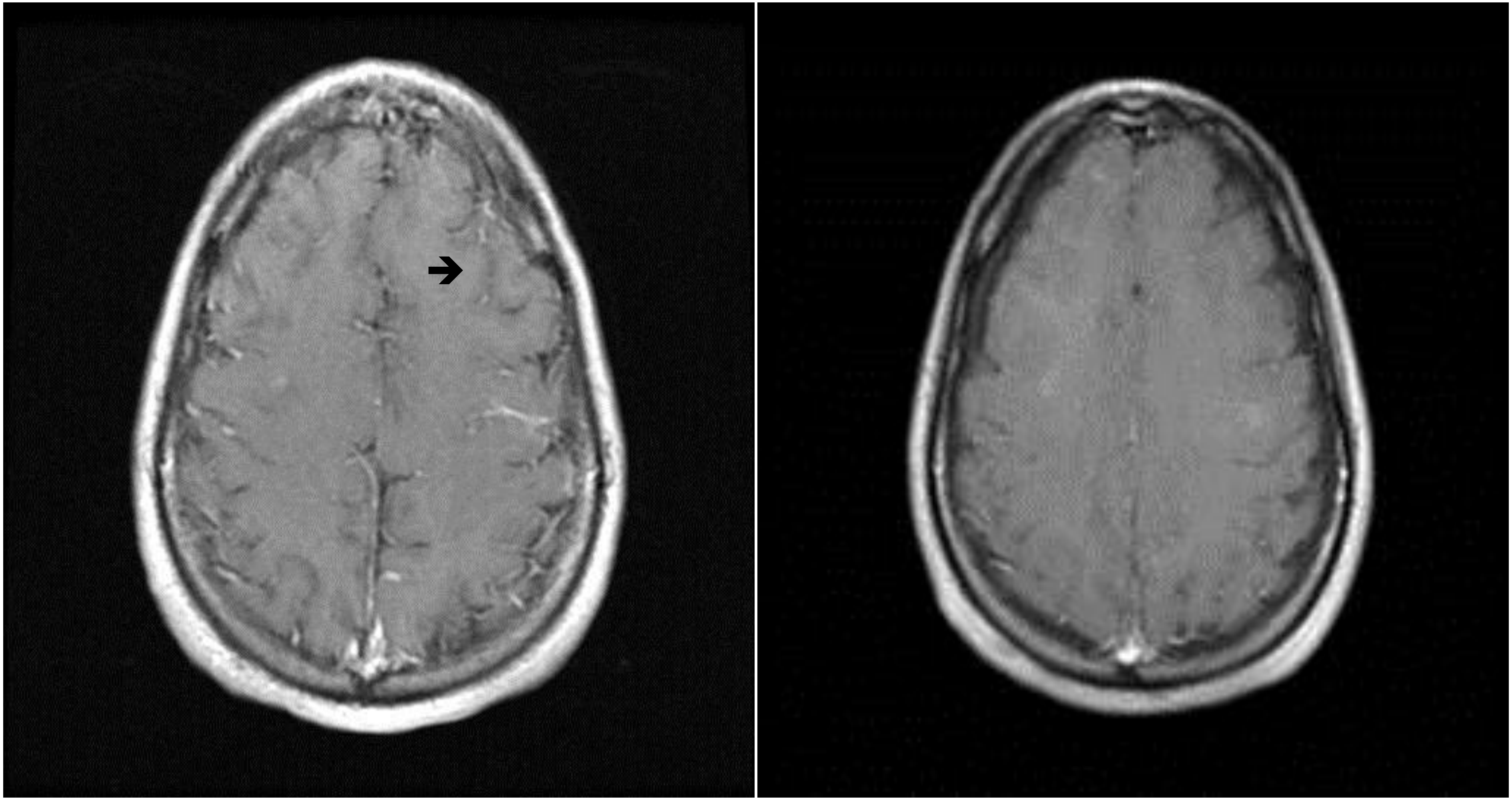

(Image Six)

Patient Two – A: MRI imaging shows the T1 post-contrast image demonstrating the cord·
lesion in the left frontal lobe in 12/2010. B: On repeat imaging in 07/2011, the enhancem
mostly resolved.
